# Anticipated Motives for Gambling Treatment in Adults from the U.S.

**DOI:** 10.1007/s10899-024-10287-6

**Published:** 2024-02-24

**Authors:** Jennifer T. Grant Weinandy, Alexander Connolly, Christopher Floyd, Shane W. Kraus, Joshua B. Grubbs

**Affiliations:** 1 Department of Psychology, Ohio University, Athens, Ohio USA; 2 Department of Psychology, University of New Mexico, Albuquerque, New Mexico USA; 3 Department of Psychology, Bowling Green State University, Bowling Green, Ohio USA; 4 Department of Psychology, University of Nevada, Las Vegas, Las Vegas, Nevada USA; 5 Department of Psychology, Center on Alcohol, Substance use, and Addictions, University of New Mexico, Albuquerque, New Mexico USA

**Keywords:** Problem gambling, Treatment seeking, Motives, Responsible gambling

## Abstract

Prior works note that identifying problematic play is a leading barrier to treatment seeking, contributing to low treatment rates in those with gambling problems (e.g., Bijker et al., 2022; Suurvali et al., 2012a). While research has looked at treatment seekers’ motives for treatment (Gainsbury et al., 2014; Suurvali et al., 2012b), the situations or signs (anticipated motives) individuals look for that suggest they would need treatment in the future remains unknown. Participants were gamblers (*N* = 1,791) from a census-matched U.S. sample of adults who reported no concerns about their gambling. Participants completed questions assessing how much money they would have to lose while gambling to think they might have a problem and what factors might motivate them to pursue treatment for gambling problems in the future. Participants reported a wide range of financial loss that would suggest they had a problem, and higher income men who gambled more frequently reported higher necessary losses. There was little variation in endorsement (40–60%) of 14 situations that may lead them to seek treatment in the future (e.g., felt guilty). However, income, gender identity, and problem gambling behavior were linked to the endorsement of some of these anticipated motives, with some differences in endorsement between those engaging in high- and low-level problem gambling. Collectively, results are consistent with the inference that many individuals may not be aware of what problematic gambling would look like for them, though income, gender identity, and problem gambling behavior may impact their consideration of anticipated motives.

**Supplementary Information:**

The online version contains supplementary material available at 10.1007/s10899-024-10287-6.

Gambling Disorder is increasingly present in the public awareness in the U.S., due in large part to expansions in access to gambling over the past decade (Welte et al., 2015). This rapid legalization of gambling is particularly concerning given that much of this newfound access is via online gambling and sports wagering, both of which are especially related to problem gambling (Allami et al., 2021; Binde, 2011; Chóliz, 2016; Grubbs & Kraus, 2022, 2023). Perhaps even more troubling, only about 4% of people with moderate levels of problem gambling ever seek treatment (Bijker et al., 2022). The leading barrier to treatment appears to be identifying problematic gambling. That is, people seem to be unaware that they even have problems, which precludes treatment seeking. This knowledge deficit is a primary target for responsible gambling (RG) initiatives, which includes activities such as wagering within affordable limits, being educated about gambling harms, and utilizing strategies such as deposit limits and self-exclusion (Blaszczynski et al., 2011; Gainsbury & Blaszczynski, 2020; Winters & Derevensky, 2020). Despite such efforts, there remains relatively little research examining what sorts of problems might lead a recreational gambler to suspect they have a gambling problem or to seek help. That is, though RG initiatives certainly attempt to make people more aware of the warning signs for problem gambling, how the general public thinks about their own gambling behaviors and the potential warning signs they might look for is poorly understood. This gap is the primary impetus for the present study.

## Problem Gambling and Responsible Gambling

The American Psychiatric Association’s *Diagnostic and Statistical Manual of Mental Disorders-Fifth Edition* (DSM-5; APA, 2013) defines Gambling Disorder as ‘persistent and recurrent problematic gambling behavior leading to clinically significant impairment or distress.’ Yet, on a broader level, both clinical and subclinical (i.e., people meeting some criteria for Gambling Disorder, but not all) levels of problematic gambling can be referred to as problem gambling. Problem gambling is broadly defined as gambling that has adverse consequences for the self, families, or communities, including financial distress, decreased well-being, relationship issues, negative mental and physical health outcomes, and negative impacts on significant others (Browne & Rockloff, 2018; Langham et al., 2016; Loo et al., 2019; Neal et al., 2005). Problem gamblers spend a significantly higher number of days gambling and gamble four-to-five times more money than those who do not experience problem gambling (Allami et al., 2021; Orford et al., 2013). Problem gambling behavior is also associated with an increased risk of developing Gambling Disorder, or pathological or compulsive gambling (Blaszczynski et al., 2011), such that it has now been labeled a public health issue (John et al., 2020).

Given that those with gambling problems may be at an increased risk for Gambling Disorder, it is important to consider ways to prevent problem gambling and encourage those who need treatment to seek it (Binde, 2011; Williams et al., 2008). RG strategies and programs have been proposed as one means of both supporting those with problem gambling and preventing gambling problems. These strategies involve a broad range of social responsibility initiatives and practices, such as providing education on the harms of problem gambling, setting maximum bet sizes, and other harm reduction and minimization behaviors that an individual can implement (Blaszczynski et al., 2011). RG practices have been shown to be effective in reducing rates of problem gambling and are recommended for all gamblers, not just those with problems (Wood & Griffiths, 2015). These RG practices encourage gamblers to think about the consequences of their gambling and limit the amount of money and time that is spent on gambling (Ladouceur et al., 2016).

Despite the increasing prominence of RG in the literature, prior work indicates that about 50% of gamblers were not aware of RG/harm reduction strategies (Christensen et al., 2022) and many gamblers who need treatment do not receive it (Bijker et al., 2022). In fact, we still know very little about how many people use these practices. Surveys conducted in the United Kingdom indicate that only 8% of gamblers have used limit setting, a specific RG practice involving voluntarily placing time or money limits on the amount one gambles (Gainsbury & Blaszczynski, 2020; Gambling Commission, 2021; Ladouceur et al., 2017). Likewise, only 43% of those surveyed were aware of the most widely used RG technique, self-exclusion, which involves the gambler excluding themselves from the casino or website in which they gamble (Gambling Commission, 2021; Ladouceur et al., 2017; Motka et al., 2018). Additionally, prior works suggest that a leading barrier to seeking treatment for problem gamblers is difficulty identifying gambling problems (Suurvali et al., 2009). Thus, in addition to education about RG habits, an effective RG program ought to provide information to gamblers about seeking treatment, given the current low rates of treatment-seeking in individuals with problem gambling (Blaszczynski et al., 2007, 2011).

## Barriers to and Motivators for Gambling-Related Treatment

Prior works show that problem gambling affects up to 4.6% of the adult population in the U.S. (Welte et al., 2015), but most gamblers who need help do not seek treatment (Bijker et al., 2022; Petry, 2005). According to two large U.S. surveys, the Gambling Impact and Behavior Study (GIBS) and the National Epidemiological Survey on Alcohol and Related Conditions (NESARC), only 7–12% of individuals reporting past Gambling Disorder had received treatment or attended a self-help group for gambling-related problems (Slutske, 2006). Further, a recent meta-analysis found even lower levels (about 4%) of individuals with moderate levels of problem gambling sought treatment (Bijker et al., 2022). Research has pointed to several environmental, systemic, and personal factors that contribute to the reduced treatment-seeking found among those engaging in problem gambling. Examples include limited availability and lack of awareness of gambling support services, lack of education about their quality and efficacy, feelings of guilt or shame, fear of stigma, and underestimation of gambling problem severity (Gainsbury et al., 2014; Suurvali et al., 2009). Likewise, research has demonstrated that some people with gambling problems are sometimes unable to recognize such issues when they arise, perhaps because they have not thought about what it may look like to gamble problematically and need to seek treatment (Jindani et al., 2021; Suurvali et al., 2012a).

In general, studies indicate that common motives for seeking treatment include financial difficulties or concerns, psychological or physical health issues, relationship issues, and work or legal difficulties (Lischer et al., 2023; Pulford et al., 2009; Suurvali et al., 2010). Research on factors associated with seeking treatment for gambling problems also indicate that the severity of gambling problems, greater problem awareness, and external pressure may facilitate treatment seeking (Braun et al., 2014; Wieczorek & Dąbrowska, 2018). Of these motives, research suggests that the most common motives noted by people who have sought help are financial difficulties and relationship issues (Suurvali et al., 2012a). However, these studies focus on those who have already sought treatment, and it is unclear if these motives are being considered prior to developing problems and seeking treatment.

Given that such a small proportion of individuals with Gambling Disorder seek or enter treatment and identifying the problem is a leading barrier, there is a need for research examining motives for help-seeking behavior before such problems may arise. Specifically, most of the previously noted reasons for help-seeking map onto the diagnostic criteria for Gambling Disorder (e.g., has made repeated unsuccessful efforts to control, cut back, or stop gambling) suggesting that these could also be anticipated motives for future treatment-seeking. However, the anticipated motives and situations that gamblers believe would spur them into treatment in the future remain unknown.

## Present Study

As previously mentioned, many individuals who report problematic gambling behavior do not seek treatment and are unable to identify these problems when they arise (Bijker et al., 2022; Suurvali et al., 2009, 2012a). Prior work has identified barriers that contribute to the reduced treatment-seeking and motives for seeking treatment in those who have already sought treatment (Gainsbury et al., 2014; Jindani et al., 2021; Lischer et al., 2023; Pulford et al., 2009; Suurvali et al., 2010, 2012b). However, for individuals who do not believe they are experiencing gambling problems, the anticipated reasons for future treatment-seeking if problems were to occur remain unknown.

As such, the present study had three specific aims: (1) to identify whether people are thinking about any of the DSM-5 diagnostic criteria for Gambling Disorder as a way to identify if they may have a problem in the future, (2) to understand whether age, gender, income, gambling frequency, disapproval of gambling, and problem gambling behavior were related to these anticipated motives, and (3) to understand if there were differences between gamblers engaging in moderate to high and no to low levels of problem gambling behaviors in terms of anticipated motives. In achieving these aims, we gain insights into whether gamblers are aware of potential signs of problem gambling and need for treatment, suggesting future avenues for responsible play initiatives and increasing treatment engagement.

## Method

### Participants and Procedure

This study included a sample of participants selected from a larger study on gambling in March 2022 (see Grubbs & Kraus, 2022). Participants were recruited via YouGov opinion polling, an international polling firm that uses a sample-matching method to construct census-matched samples. YouGov has been shown to outperform other probability and non-probability vendors with regards to accuracy and representativeness (Kennedy et al., 2016; Rivers, 2016) and past work has clearly shown that YouGov is useful for studying gambling behavior (Sturgis & Kuha, 2021, 2022). For the present data, YouGov drew on a random hypothetical sample based on the 2019 American Community Survey (ACS) that corresponds to the sampling frame and selects panelists who match sampling frame members’ demographic characteristics. The matched cases were then weighted to the sampling frame using propensity scores. YouGov provided poststratification weights, based on age, race, ethnicity, educational level, and both 2020 and 2016 vote history, when available. Based on their proprietary data collection standards, specific measures of quality control and responsiveness were guaranteed as a part of YouGov’s data collection process.

Of the sample of adults matched to U.S. national norms (*N* = 4,363), we focused on those with a history of at least one gambling behavior (*N* = 4,066) and then selected participants who reported that they did not believe they had a problem with out-of-control gambling or were unsure if they had a problem (*N* = 1,791). All participants were asked to complete all the measures detailed below in the online survey during a data collection in 2022, and only those who responded to all anticipated motives questions were included. Most of the participants were men (58.5%), with a past-year history of at least one gambling behavior (99.5%), a modal income of $60,000 to $69,000 annually, and a mean age of 51 years (*SD* = 15 years). See Table 1 for full demographic information for these participants.

**Table 1:** Demographics and problem gambling severity levels

Variable	N	%
Gender		
Female	710	39.6%
Male	1047	58.5%
Non-binary	21	1.2%
Other	13	0.7%
Mean Age	50.78 (*SD* = 15.36)	
Race/Ethnicity		
White	1258	70.2%
Black	196	11.0%
Hispanic	176	9.8%
Asian	53	3.0%
Native American	26	1.5%
Two or more races	40	2.2%
Other	28	1.6%
Middle Eastern	13	0.7%
Marital Status		
Married	950	53.0%
Separated	37	2.1%
Divorced	208	11.6%
Widowed	78	4.4%
Never married	416	23.2%
Domestic/civil partnership	102	5.7%
Education		
No HS	43	2.4%
High school graduate	422	23.6%
Some college	333	18.6%
2-year	211	11.8%
4-year	475	26.5%
Post-grad	307	17.1%
Problem Gambling Severity		
High problem gambling	243	13.4%
Moderate problem gambling	106	5.9%
Low problem gambling	531	29.8%
No problem gambling	906	50.7%
Median Family Annual Income	$60,000-$69,999	

Note: *N* = 1,791

### Measures

Three gambling-specific measures were used to assess gambling: Frequency, disapproval, and problems experienced with gambling. Gambling frequency was measured by asking participants how often they gambled over the past 12 months, with scores ranging from 1 (*Never*) to 7 (*More than once a day*). Participants’ mean self-reported gambling frequency was 2.92 (*SD* = 1.21), which corresponded most closely to a monthly frequency. Disapproval of gambling was measured by asking participants their agreement with the statement “I believe that gambling is morally wrong.” on a 7-point scale ranging from 1 (*Strongly disagree*) to 7 (*Strongly agree*). See Table 2 for means and standard deviations.

**Table 2:** Correlations between anticipated motives and demographics

Anticipated Motives	Age	Gender	Income	Frequency	Disapproval	PGSI
Loved One Voiced Concern	− 0.047^*^	− 0.001	0.094^**^	− 0.004	− 0.023	− 0.025
Mental Health Professional Voiced Concern	− 0.057^*^	− 0.054^*^	0.113^**^	− 0.012	− 0.043	− 0.036
Doctor Voiced Concern	-0.04	− 0.048^*^	0.107^**^	− 0.031	− 0.021	− 0.068^**^
Lost More Money Than Intended	− 0.055^*^	− 0.035	0.025	0.033	0.045	0.086^**^
Felt You Couldn’t Stop	0.006	− 0.03	0.092^**^	0.024	− 0.079^**^	− 0.051^*^
Problems at Work	− 0.033	− 0.050^*^	0.115^**^	− 0.025	− 0.043	− 0.078^**^
Lying to Conceal Gambling	− 0.008	− 0.043	0.067^**^	− 0.009	− 0.035	− 0.044
Felt Guilty	− 0.075^**^	− 0.057^*^	0.050^*^	0.025	0.077^**^	0.114^**^
Needed to Bet More Money	0.014	− 0.037	0.066^**^	0.018	− 0.025	− 0.024
In Debt to Gamble	0.001	− 0.016	0.099^**^	0.005	− 0.072^**^	− 0.062^**^
Relationship Problems	− 0.009	− 0.015	0.113^**^	0.012	− 0.049^*^	− 0.053^*^
Tried and Couldn’t Stop	0.007	− 0.033	0.082^**^	0.013	− 0.058^*^	− 0.061^**^
Gambling to Cope	− 0.013	− 0.038	0.070^**^	0.036	0.013	0.029
Experienced an Urge or Cravings	0.011	− 0.049^*^	0.068^**^	0.019	0.022	0.04
Amount of Money Lost	− 0.020	0.144**	0.292**	0.166**	− 0.180**	− 0.003
*M*	50.78	0.58	7.29	2.92	2.49	0.30
*SD*	15.36	0.49	3.52	1.21	1.65	0.54

Note: *N* = 1,791; PGSI = Problem Gambling Severity Index; Gender: Male = 1, Not Male = 0; Amount of Money Lost was entered as Log10(x + 1) transformed; * *p* ≤ .05, ** *p* ≤ .01

Problems experienced with gambling were measured using the Problem Gambling Severity Index (PGSI), which asks participants to rate their agreement with several statements about problems they may experience due to gambling on a scale from 1 (*Never*) to 4 (*Almost always*). This scale has previously been shown to be a reliable measure of problem gambling (Holtgraves, 2009) and obtained an Omega Total of 0.94 in this study. Scores on this measure were averaged and ranged from 0 to 3, with this study’s participants obtaining a mean score of 0.31 (*SD* = 0.54). The PGSI can also be used to group participants into no problem gambling behavior, low problem gambling behavior, moderate problem gambling behavior, and high problem gambling behavior groups using the total PGSI score (Currie et al., 2013). To obtain the sum in accordance with the standard cut-offs, we transformed the responses to a 0 (*Never*) to 3 (*Almost always*) scale and summed participants’ responses. In this study, 243 participants (13.4%) were considered engaging in high levels of problem gambling (scoring 8 or more), 106 participants (5.9%) were considered engaging in moderate levels of problem gambling (scoring between 5 and 7), 531 participants (29.7%) were considered engaging in low levels of problem gambling (scoring between 1 and 4), and 906 participants (50.7%) were considered engaging in no problem gambling (scoring 0).

Anticipated motives for treatment for gambling problems were assessed by asking participants the degree to which 14 circumstances (e.g., felt guilty after gambling) would indicate that they should seek treatment for a gambling problem in the future. Agreement with each of these statements were rated on a scale from 1 (*Not at all likely*) to 3 (*Very likely*). For a full list of these items, see Online Resource 1. See Table 3 for means and standard deviations for agreement on each statement. One additional item asked participants how much money they would need to lose to indicate that they have a problem with gambling and should seek treatment (final anticipated motive). Participants’ average amount that they believed they would need to lose was $18, 311 (*SD* = $337, 443), ranging from $0 to $10,000,000 with a median of $1,000. Given that this item was significantly skewed, this item was transformed using the Log10(*x* + 1) function for all analyses.

### Analyses

To respond to the aims of this study, four types of analyses were used. First, multiple chi-squared analyses were conducted to understand the differences in endorsement of anticipated motives between those who selected not at all likely and those that selected either Somewhat or Very Likely to suggest a need for treatment. In other words, a chi-squared analysis was conducted for each of the anticipated motives to understand differences in endorsement between these two groups.

Next, Pearson Product-Moment correlations were conducted to understand the relationships between each of the anticipated motives. Further correlations were also conducted to understand the relationships between these anticipated motives and each of the demographics (age, gender, and income) and gambling-specific measures (gambling frequency, disapproval, and problems experienced).

Following this, we conducted multiple linear regression analyses to understand the impact of the demographic and gambling-specific measures on the rate of endorsement of each of the anticipated motives, resulting in 15 regression analyses. In this way, the demographic and gambling-specific measures were entered as predictor variables and each of the anticipated motives were added as an outcome variable in one of these regressions.

Finally, we conducted 14 chi-squared analyses to understand the differences in endorsement of each of the anticipated motives (*Not at all likely* vs. *At least somewhat likely*) between those who were identified as no-to-low-level problem gamblers and moderate-to-high-level problem gamblers on the PGSI. We did not conduct a chi-squared analysis between these groups for the anticipated motive of the amount of money lost, given that it was a continuous variable which would have been inappropriate to dichotomize.

## Results

To better understand the anticipated reasons for treatment seeking in the future in individuals who do not believe they have problems with gambling behavior, we investigated: (1) the endorsement of several potential anticipated motives for treatment, (2) whether key demographic and gambling related behaviors were related to these anticipated motives, and (3) whether there are differences between gamblers engaging in high- and low-level problem gambling behavior in terms of endorsement of anticipated motives.

### Little Variation Between Endorsement of Anticipated Motives

**Fig. 1:**
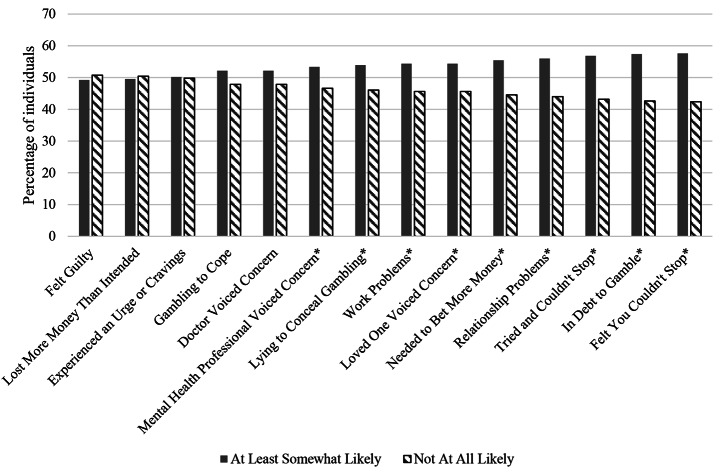
Percentages of participants who endorsed each anticipated motive as a reason to seek treatment in the future, with the results of a χ^2^ test comparing levels of endorsement *Note:N* = 1,791. * Significant *χ*^*2*^ test result with a *p*-vaue of < 0.05. Results suggest that endorsement of each motive is relatively equal, lying between 40% and 60% of participants. However, more people endorsed nine of these anticipated as at least somewhat likely to lead them to treatment in the future than not at all likely, suggesting that some may be more important than others

To understand whether there were differences in endorsement between the anticipated motives, we conducted multiple chi-squared analyses between those who endorsed the motive as at least somewhat, compared to not at all, likely to indicate the need for treatment (See Fig. 1 for an overview of these results). Visual inspection suggests slight variation between the percentages of participants who endorsed each of the 14 anticipated motives (between 40 and 60%). However, significantly more participants endorsed the following anticipated motives as at least somewhat, compared to not at all, likely to indicate a need for treatment: Loved One Voiced Concern, Mental Health Professional Voiced Concern, Problems at Work, Relationship Problems, Felt You Couldn’t Stop, Tried and Couldn’t Stop, Needing to Bet More Money, Lying to Conceal Gambling, and In Debt to Gamble. These results suggest that gamblers are not thinking of one reason to seek treatment in the future, but that experiencing difficulty stopping, betting more over time, and some interpersonal, financial, or work consequences may be more often considered as reasons.

To understand how the endorsement of each of the anticipated motives were related to each other, we conducted correlations between these motives (See Table 3). Each of the anticipated motives were highly correlated with one another (*r* > .543), except for Amount of Money Lost. The Amount of Money Lost anticipated motive showed nonsignificant correlations with all other anticipated motives except the Tried and Couldn’t Stop and the Gambling to Cope anticipated motives (*r* = .046 and 0.051 respectively). As such, these results suggest that the anticipated motives may reflect a similar construct and are seen as similar in terms of how likely they are to suggest a need for treatment, except for Amount of Money Lost.

**Table 3:** Correlations between each of the anticipated motives alongside their means and standard deviations

	1.	2.	3.	4.	5.	6.	7.	8.	9.	10.	11.	12.	13.	14.		15.
1. Loved One Voiced Concern	1															
2. Mental Health Professional Voiced Concern	0.721^**^	1														
3. Doctor Voiced Concern	0.730^**^	0.816^**^	1													
4. Lost More Money Than Intended	0.602^**^	0.554^**^	0.557^**^	1												
5. Felt You Couldn’t Stop	0.726^**^	0.727^**^	0.714^**^	0.578^**^	1											
6. Problems at Work	0.704^**^	0.763^**^	0.746^**^	0.584^**^	0.750^**^	1										
7. Lying to Conceal Gambling	0.717^**^	0.720^**^	0.708^**^	0.618^**^	0.759^**^	0.766^**^	1									
8. Felt Guilty	0.598^**^	0.561^**^	0.543^**^	0.600^**^	0.577^**^	0.556^**^	0.622^**^	1								
9. Needed to Bet More Money	0.694^**^	0.723^**^	0.714^**^	0.635^**^	0.770^**^	0.748^**^	0.754^**^	0.601^**^	1							
10. In Debt to Gamble	0.700^**^	0.739^**^	0.726^**^	0.584^**^	0.806^**^	0.780^**^	0.761^**^	0.547^**^	0.752^**^	1						
11. Relationship Problems	0.747^**^	0.751^**^	0.751^**^	0.581^**^	0.797^**^	0.801^**^	0.784^**^	0.573^**^	0.747^**^	0.798^**^	1					
12. Tried and Couldn’t Stop	0.729^**^	0.744^**^	0.742^**^	0.577^**^	0.813^**^	0.763^**^	0.766^**^	0.587^**^	0.775^**^	0.791^**^	0.801^**^	1				
13. Gambling to Cope	0.652^**^	0.657^**^	0.661^**^	0.605^**^	0.671^**^	0.690^**^	0.706^**^	0.619^**^	0.704^**^	0.669^**^	0.676^**^	0.696^**^	1			
14. Experienced an Urge or Cravings	0.647^**^	0.624^**^	0.617^**^	0.589^**^	0.661^**^	0.656^**^	0.680^**^	0.645^**^	0.699^**^	0.627^**^	0.627^**^	0.665^**^	0.685^**^	1		
15. Amount of Money Lost	0.036	0.043	0.033	− 0.007	0.042	0.032	0.021	− 0.001	0.043	0.033	0.039	0.051*	0.046*	0.016		1
*M*	1.76	1.82	1.79	1.69	1.93	1.88	1.80	1.66	1.86	1.98	1.89	1.91	1.75	1.71		2.87
*SD*	0.79	0.85	0.84	0.78	0.88	0.88	0.83	0.75	0.86	0.91	0.87	0.87	0.80	0.79		0.86

Note. *N* = 1,791; SD = Standard Deviation; M = Mean; Amount of Money Lost was entered as Log10(x + 1) transformed; ^*^Correlation is significant at the 0.05 level; ^**^Correlation is significant at the 0.01 level

### Predictors and Correlates of Amount of Money Lost

Given that the Amount of Money Lost anticipated motive appeared mostly unrelated to the other anticipated motives and can be conceptualized uniquely as a continuous variable (i.e., an amount of money they would need to lose vs. a dichotomous endorsement of whether a situation is problematic), the decision was made to discuss this anticipated motive first.

To understand whether demographics (i.e., age, gender, and income) and gambling related variables (i.e., gambling frequency, gambling disapproval, and problem gambling behavior) were related to and predicted the Amount of Money Lost anticipated motive, we first conducted correlation analyses (See Table 2) followed by a linear regression analysis, with the demographic and gambling related variables entered as predictors (See Table 4). Identifying as male, income, and gambling frequency showed small, positive correlations with (*r* = .144 to 0.292) and positively predicted Amount of Money Lost. Disapproval of gambling was also negatively predicted and achieved a small, negative correlation (*r* = − .180) with Amount of Money Lost. These results suggest that those who identify as male and have higher income, higher gambling frequency, and less disapproval of gambling are more likely to believe that they would need to lose more money to signify a problem.

**Table 4:** Regression table between anticipated motives and demographics

Anticipated Motives	Age β	Gender β	Income β	Disapproval β	Frequency β	PGSI β	R^2^	F
Loved One Voiced Concern	− 0.035	− 0.010	0.090***	− 0.024	− 0.013	0.012	0.011	3.28**
Mental Health Professional Voiced Concern	− 0.052*	− 0.067**	0.111***	− 0.040	− 0.009	− 0.012	0.021	6.35***
Doctor Voiced Concern	− 0.039	− 0.058*	0.108***	− 0.011	− 0.022	− 0.033	0.018	5.39***
Lost More Money Than Intended	− 0.020	− 0.037	0.028	0.016	0.014	0.087**	0.013	3.96***
Felt You Couldn’t Stop	0.003	− 0.049*	0.090***	− 0.066**	0.028	− 0.021	0.016	4.96***
Problems at Work	− 0.041	− 0.063**	0.115***	− 0.024	− 0.010	− 0.059*	0.022	6.82***
Lying to Conceal Gambling	− 0.002	− 0.052*	0.070**	− 0.032	− 0.007	0.002	0.008	2.46*
Felt Guilty	− 0.023	− 0.058*	0.059*	0.039	0.000	0.118***	0.027	8.35***
Needed to Bet More Money	0.025	− 0.049*	0.071**	− 0.021	0.017	0.012	0.008	2.31*
In Debt to Gamble	− 0.002	− 0.032	0.096***	− 0.059*	0.006	− 0.021	0.015	4.56***
Relationship Problems	− 0.005	− 0.031	0.112***	− 0.037	0.011	− 0.014	0.015	4.68***
Tried and Couldn’t Stop	0.005	− 0.048*	0.082***	− 0.045	0.019	− 0.022	0.012	3.60**
Gambling to Cope	0.018	− 0.049*	0.077**	− 0.001	0.021	0.067*	0.012	3.63**
Experienced an Urge or Cravings	0.047	− 0.057*	0.081***	0.010	0.004	0.072**	0.013	4.06***
Amount of Money Lost	− 0.002	0.088***	0.260***	− 0.154***	0.126***	0.019	0.137	47.16***

Note. *N* = 1,791; PGSI = Problem Gambling Severity Index; Gender: Male = 1, Not Male = 0; Amount of Money Lost was entered as Log10(x + 1) transformed; *Significant at the 0.05 level; **Significant at the 0.01 level; ***Significant at the 0.001 level

### Predictors and Correlates of Remaining 14 Anticipated Motives

To understand whether demographics (i.e., age, gender, and income) and gambling related variables (i.e., gambling frequency, gambling disapproval, and problem gambling behavior) were related to the remaining 14 anticipated motives, we first conducted correlation analyses (See Table 2) and then linear regression analyses (See Table 4). Specifically, we conducted a separate regression analysis for each of the 14 anticipated motives presented, with the demographic and gambling related behavior variables entered as predictors.

To understand whether age, gender, or income were related to the endorsement of the anticipated motives, we conducted correlational analyses. Age obtained small, negative correlations with the following anticipated motives: Loved One Voiced Concern, Mental Health Professional Voiced Concern, Lost More Money Than Intended, and Felt Guilty (*r* = − .047 to − 0.075). Identifying as male showed small, negative correlations with the Mental Health Professional Voiced Concern, Doctor Voiced Concern, Problems at Work, Felt Guilty, and Experienced an Urge or Cravings anticipated motives (*r* = − .048 to − 0.057). Income obtained exceedingly small to small, positive correlations with all anticipated motives except Lost More Money Than Intended (*r* = .050 to 0.115). These results show that the anticipated motives were most often related to higher income, suggesting that those with a higher income may be thinking more about reasons to seek treatment for gambling.

To understand whether gambling frequency, disapproval, or problems with gambling were related to each of the anticipated motives, we conducted correlation analyses. Gambling frequency appeared unrelated to all 14 anticipated motives. Disapproval of gambling and PGSI score showed very small to small, positive correlations with the Felt Guilty anticipated motive (*r* = .077 and 0.114) and very small, negative correlations with the Felt You Couldn’t Stop, In Debt to Gamble, Relationship Problems, and Tried and Couldn’t Stop anticipated motives (*r* = − .049 to − 0.079). Further, PGSI score obtained small, positive correlations with Lost More Money Than Intended (*r* = .086) and small, negative correlations with Doctor Voiced Concern and Problems at Work (*r* = − .068 and − 0.078). These results suggest that gambling disapproval and problems experienced, but not frequency, are more often related to considering anticipated motives for treatment.

To understand whether any of the demographic variables predicted the endorsement of each of the anticipated motives, we conducted multiple linear regression analyses with each of the anticipated motives. Age negatively predicted the Mental Health Professional Voiced Concern anticipated motive, such that younger individuals believed they would be more motivated by a Mental Health Professional speaking with them than older individuals. Identifying as male negatively predicted all anticipated motives except Loved One Voiced Concern, Lost More Money Than Intended, In Debt to Gamble, and Relationship Problems. Income positively predicted all anticipated motives except Lost More Money Than Intended. These results suggest that gender identity likely impacts consideration of anticipated motives and those with higher income consider many of the anticipated motives as reasons to seek treatment.

To understand whether gambling frequency, disapproval, or problems were related to each of the anticipated motives, we again examined the multiple regression analyses. Frequency did not significantly predict any of the 14 anticipated motives for treatment. Disapproval of gambling negatively predicted the Felt You Couldn’t Stop and In Debt to Gamble, anticipated motives, such that the less disapproval of gambling a participant had, the more likely they were to endorse these motives. PGSI positively predicted the Lost More Money Than Intended, Felt Guilty, Gambling to Cope, and Experienced an Urge or Cravings anticipated motives, and negatively predicted the Problems at Work anticipated motive. These results suggest that disapproval of gambling and experiencing gambling problems may be more predictive of thinking about anticipated motives for treatment than the frequency of gambling behavior.

Notably, the amount of variance explained by the demographic and gambling-specific predictor variables for these 14 anticipated motives’ regression analyses remained relatively low (*R*^*2*^ = 0.008 to 0.027). This suggests that these variables may not be the best predictors of these anticipated motives for gambling treatment.

### Differences Between High- and Low-Level Problem Gamblers

**Fig. 2:**
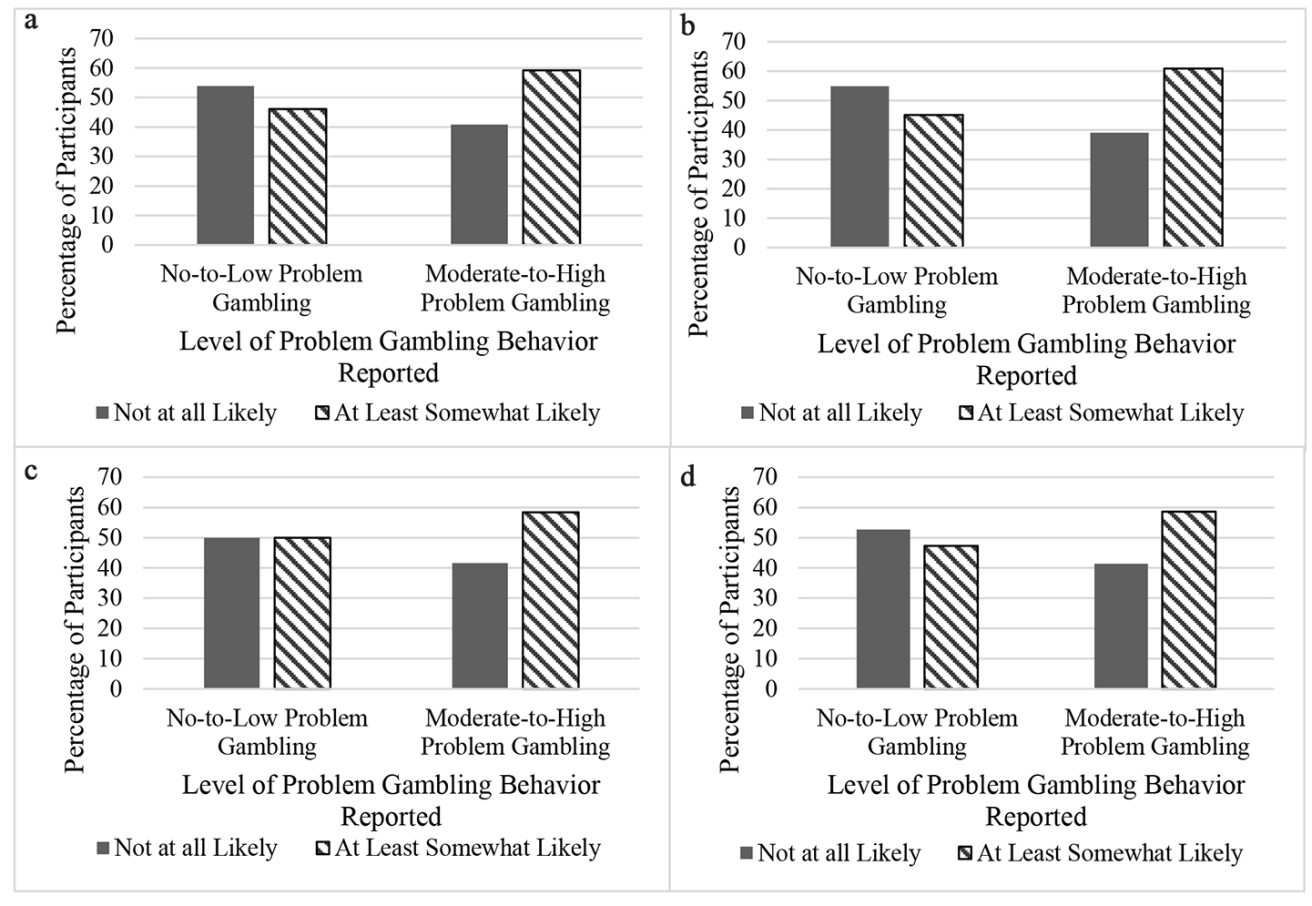
Percentage of participants engaging in no to low problem gambling behavior and moderate to high problem gambling behavior who endorsed lost more money than intended, felt guilty, gambling to cope, and experienced an urge or cravings as anticipated motives. **a.** Lost More Money Than Intended. **b.** Felt Guilty. **c.** Gambling to Cope. **d.** Experienced an Urge or Cravings *Note:N* = 1,791. All analyses shown obtained significant *χ*^*2*^ test result with a *p*-vaue of < 0.05. Percentage of participants who endorsed Lost More Money Than Intended (**a**), Felt Guilty (**b**), Gambling to Cope (**c**), and Experienced an Urge or Cravings (**d**) as reasons to seek treatment in the future. Results suggest that those who reported moderate-to-high-level problem gambling behavior more highly endorsed these anticipated motives as potential reasons to seek treatment more than those who reported no-to-low-level problem gambling behavior

To understand whether there were differences in endorsement of the 14 anticipated motives between those who engaged in moderate-to-high levels of problem gambling, compared to no-to-low-levels, we conducted multiple chi-squared analyses (See Fig. 2 and Online Resource 2). There were significant differences between these problem gambling behavior groups in terms of endorsement for the Lost More Money Than Intended (*χ*^*2*^ = 24.00, *p* = .000), Felt Guilty (*χ*^*2*^ = 34.81, *p* = .000), Gambling to Cope (*χ*^*2*^ = 9.86, *p* = .002), and Experienced an Urge or Cravings (*χ*^*2*^ = 17.71, *p* = .000) anticipated motives. More specifically, these analyses showed that those who reported moderate-to-high-level problem gambling more highly endorsed these anticipated motives as potential reasons to seek treatment than those who reported no-to-low-level problem gambling behavior. These results suggest that those who engage in more problem gambling behavior consider losing more than they intend, feeling guilty, using gambling to cope, and craving gambling as indicators of a need to get treatment more than those who engage in lower levels of problem gambling.

## Discussion

The motives and situations that gamblers believe would spur them into treatment in the future remain unknown. In this paper, we found limited variation of endorsement between the presented anticipated motives based on Gambling Disorder DSM-5 criteria, suggesting that there is not one specific anticipated motive that individuals expected to signal a need for treatment. Further, the amount of money individuals believed they would need to lose to suggest they had a problem appeared mostly unrelated to other anticipated motives. This Amount of Money Lost anticipated motive was predicted by income, gender identity, gambling frequency, and gambling behavior. While age, gender identity, and gambling frequency were related to and predicted the endorsement of some of the remaining 14 anticipated motives, income and problem gambling behavior were the only relatively consistent predictors of endorsement. Compared to those engaging in no to low-level problem gambling behaviors, those with higher level problem gambling behavior appeared to endorse Lost More Money Than Intended, Felt Guilty, Gambling to Cope, and Experienced an Urge or Cravings as potential anticipated motives more often. Overall, these results suggest that many gamblers are not thinking about what problematic gambling may look like for them, but income, gender identity, and problem gambling behavior may impact their consideration of anticipated motives.

Research shows a lack of awareness of RG initiatives, such as self-exclusion, (Christensen et al., 2022; Gambling Commission, 2021; Ladouceur et al., 2017; Motka et al., 2018) despite also showing that these practices may be helpful in reducing problem gambling for all gamblers (Wood & Griffiths, 2015). These results highlight that lack of awareness of what problematic gambling would look like for the individual, which is likely a reason for the previously cited difficulties individuals experience in terms of recognizing problematic gambling and a need to seek treatment (Bijker et al., 2022; Jindani et al., 2021; Suurvali et al., 2009). Specifically, our results show that the amount of money an individual believes they need to lose was unrelated to the other anticipated motives, suggesting that these gamblers often did not consider these concepts in the same way in terms of signifying the need for treatment. Similarly, endorsement rates found in this study suggest that there is a 40–60% chance of endorsement of any of the anticipated motives, indicating that individuals have not been considering any of these anticipated motives as potential reasons to seek treatment. Presumably, if these gamblers are not considering these anticipated motives, then they will be unaware when they begin to show early signs of problematic gambling. As such, they may not recognize a problem when it begins to occur, leading to increased harm over time and likely a lack of awareness that they may benefit from treatment.

Individual demographic factors may still impact consideration of anticipated motives, and therefore potentially engagement in RG behavior. Prior work suggests that those in lower socioeconomic groups make decisions based on immediate need, whereas those in higher socioeconomic groups more often consider longer-term outcomes (Sheehy-Skeffington, 2020). This may explain our results showing that higher income predicted more endorsement of most anticipated motives, in that those with lower income were less likely to consider future problems due to socioecological cues which force them to focus on addressing immediate needs. There are known differences in terms of gender for gambling behavior and problem gambling, such as preference for game type and mental health correlates (Baggio et al., 2018). These differences may have also impacted the effect of gender identity on endorsement of many of the anticipated motives in this study, such as using gambling to cope.

Relatedly, gambling beliefs and behaviors appear to impact consideration of anticipated motives, particularly in terms of disapproval of gambling and problem gambling behaviors. Previous work has noted that disapproval of gambling may impact beliefs about problematic gambling behavior and the need for treatment or help through moral incongruence (Grubbs et al., 2022) and stigma (e.g., Andrà et al., 2022; Delfabbro et al., 2022). Notably, our results highlight that disapproval, but not frequency, predicted endorsement of five of the anticipated motives, suggesting that these may be important pathways through which disapproval of gambling impacts engagement in RG.

Problem gambling behavior is related to higher gambling frequency and spending (Allami et al., 2021; Orford et al., 2013) and our results suggest that the problems experienced may be particularly important in terms of encouraging consideration of anticipated motives. Despite reporting that they did not have problems with gambling, a portion (13.4%) of our sample’s scores on a measure of problem gambling suggested that they did engage in higher levels of problem gambling behavior. Problem gambling behavior, but not frequency of gambling, also predicted multiple anticipated motives, suggesting that the negative consequences experienced may increase an individual’s consideration of reasons to seek treatment. This finding is encouraging because it suggests that experiencing gambling problems may heighten awareness of a need for treatment, which reflects findings in prior work (Braun et al., 2014) and could inform public health initiatives.

When comparing individuals who reported higher- and lower-level problem gambling, our results suggested that those who engaged in more problem gambling behaviors may also be more attuned to losing more money than they intended, feeling guilty, using gambling to cope, and interpreting cravings to gamble as indicators of a need for treatment. Prior work has noted that increased problem gambling behavior may facilitate treatment seeking (Braun et al., 2014), and our results indicate some areas where such facilitation may occur. However, the level of problem gambling was not related to the consideration of the amount of money the individual would need to lose, even though results showed that those who gambled more frequently suggested that they would need to lose more money. These results highlight increasing consideration of the amount of money someone could lose as a major area for future RG initiatives, especially given the prevalence of financial difficulties as a common motive for treatment seeking (Suurvali et al., 2010, 2012b).

Much of the previous literature related to motives for gambling treatment show that financial and relationship difficulties are some of the more common motives for individuals who have sought treatment difficulties (Lischer et al., 2023; Pulford et al., 2009; Suurvali et al., 2010, 2012b). This is perhaps surprising because three of the four anticipated motives (Felt Guilty, Gambling to Cope, and Experienced an Urge or Cravings) endorsed by those with higher-level problem gambling are not financial or interpersonal in nature. Further, even though prior work has highlighted problems at work as a common motive for seeking treatment (Pulford et al., 2009; Suurvali et al., 2010), those with higher levels of problem gambling were less likely to consider this as a reason for future treatment seeking in our sample. Concerningly, this suggests that the anticipated motives for treatment seeking may not actually match motives for treatment seeking in those who seek treatment. This indicates that those engaging in problem gambling behavior may not be aware of which factors would motivate them to seek treatment, likely leading to less treatment seeking overall.

### Implications and Future Directions

The current work suggests that most gamblers are not thinking about the reasons that they may need to seek treatment for gambling in the future, demonstrating that addressing this key area in responsible play initiatives would increase engagement in treatment. Overall, this work suggests important implications in terms of research, public policy, and clinical work related to improving awareness of reasons to seek treatment for gambling problems.

Future research regarding anticipated motives for gambling treatment should focus on other potential anticipated motives and possible predictors, more diverse samples, ways to increase awareness of anticipated motives, and potential generalizability to other related problematic addictive behaviors. Qualitative methodology would be particularly beneficial in understanding other potential anticipated motives and predictors of help seeking for problem gambling. Further, consideration of other correlates of treatment seeking, such as family history of gambling problems, may help to identify gamblers who are more aware of reasons to seek treatment, and are more likely to seek treatment if problems arise. Similarly, it is likely that there are differences in terms of culture regarding anticipated motives, and future work using samples from other non-Western or historically underrepresented populations could highlight these differences. Education-based programs and longitudinal research should focus on ways to increase awareness of anticipated motives, particularly the amount of money an individual may lose, and what problematic gambling may look like. This research should also follow individuals to see whether the anticipated motives that are identified spur them to seek help if they happen in the future, or whether these motives change over time. Finally, many of the anticipated motives for problem gambling, such as Relationship Problems, could also be reasons to seek treatment for other addictive behaviors or substance use disorders, but further research is needed to better understand these potential motives.

In terms of public policy, this work highlights a need for more to be done in terms of educating gamblers about what problematic gambling looks like and reasons to seek treatment, potential target areas for such education, and public health awareness campaigns which may benefit from funding and support. Given the lack of awareness of reasons to seek treatment, campaigns and education programs should highlight not just what responsible play looks like, but also what problematic play looks like. These campaigns could also focus on encouraging reflection about limit setting or highlighting consideration of specific problematic situations, such as asking gamblers “How much money are you able to spend today?” Alternatively, these policies and campaigns could focus on areas in which higher-level problem gamblers in this study were more attuned to: losing more than they intend, feeling guilty, using gambling to cope, and experiencing cravings. Public policy should consider investing more in these education programs and research focused on improving awareness of reasons to seek treatment for gambling.

Clinically, it would be beneficial to increase screening for Gambling Disorder and gambling problems, provide more psychoeducation about these problems, and consider using treatments such as Motivational Interviewing and harm reduction techniques. There is a lack of awareness of problematic gambling behavior and the potential need to seek treatment. As such, it would be beneficial for clinicians to screen for these problems at the outset of treatment, given that some individuals may not be aware of the problematic nature of their behavior. Relatedly, clinicians are well-poised to lead psychoeducation about ways to identify and cope with gambling problems, including considering ways to implement RG and harm reduction initiatives. Psychoeducation about financial harms may be more helpful for those who identify as male, have a higher income, gamble more frequently, and disapprove less of gambling, given that these individuals suggested higher amounts of money that they would need to lose to seek treatment. Individually, it is possible that treatments such as Motivational Interviewing may be a powerful way to help clients consider reasons for seeking treatment or ways to engage in less harmful gambling practices, such as setting a limit for their spending. Support like Motivational Interviewing could be particularly helpful for lower income individuals and those who identify as male, who appeared to be less likely to consider many of the anticipated motives in this study. Further, in those with more problem gambling behavior, providers may find that considering whether they have lost more than they intended, felt guilty, used gambling to cope, or experienced cravings are more salient factors for individuals in considering treatment. It is also important to highlight that treatment is not always needed for those experiencing gambling problems and natural recovery is possible (Hodgins et al., 2022; Slutske, 2006), as such clinicians and clients should collaboratively consider options. Given our results, prevention work may also benefit from focusing on problems experienced while gambling or areas of gambling disapproval, rather than the frequency of gambling, as ways to make future gambling harms more salient.

### Limitations

There are multiple limitations of the present work, including sample biases, range of responses for the Amount of Money Lost anticipated motive, possible missing predictors, and the use of diagnostic criteria for the anticipated motives. While the sample used in this study came from a larger sample of adults matched to U.S. national norms, it may not be generalizable to other countries and cultures and does not account for unique cultural differences among groups within the U.S., such as differences based on racial identity. While the PGSI is a previously validated measure of problem gambling (e.g., Currie et al., 2013; Holtgraves, 2009; Miller et al., 2013), there have been some concerns about its discriminant validity between low and moderate problem gambling categories specifically. To partially combat this, we used updated categories as proposed by Currie et al. (2013). Results showed a wide range of responses for the Amount of Money Lost anticipated motive, with significant skew, suggesting that outliers may be present. However, removing outliers did not significantly improve the skew of the data following transformation, and it appeared important to capture the full range of responses to this question. Further, multiple other possible predictors of the anticipated motives were not included in this analysis, such as past experience of gambling treatment. Finally, this study used primarily DSM-5 diagnostic criteria for Gambling Disorder as possible anticipated motives for treatment seeking. However, it is likely that individuals may consider other factors and consequences of gambling behavior as more salient anticipated motives and further work is needed to validate these items, such as comparing to qualitative responses.

## Conclusion

Prior works have highlighted a lack of awareness about the need to seek gambling treatment and focused on motives for treatment seeking in those who have sought treatment (Bijker et al., 2022; Gainsbury et al., 2014; Suurvali et al., 2010, 2012a). However, the motives and situations that gamblers believe would spur them into treatment in the future remained unknown. This study used a large sample of adults matched to U.S. national norms to investigate variation in and predictors of 15 anticipated motives based on Gambling Disorder DSM-5 diagnostic criteria. These results showed slight variation in endorsement between anticipated motives, with gender identity, higher income, and problem gambling behavior as most consistently related to and predictive of these anticipated motives. The results also suggested that consideration of the amount of money they would need to lose to signify a problem was unrelated to their consideration of most other anticipated motives. Individuals who reported engaging in a higher level of problem gambling behavior appeared to focus more on losing more than intended, feeling guilty, gambling to cope, and experiencing cravings as potential anticipated motives than those who engaged in lower levels of problem gambling behavior. Overall, these results suggest that most gamblers are not thinking about the reasons that they may need to seek treatment for gambling in the future, demonstrating that addressing this key area in responsible play initiatives would increase engagement in treatment.

## Electronic Supplementary Material

Below is the link to the electronic supplementary material.

Supplementary Material 1

Supplementary Material 2

## Data Availability

Data will be made available upon request.
