# Supplementary material for: Anticipated Motives for Gambling Treatment in Adults from the U.S
Source: J Gambl Stud. 2024 Feb 24;40(3):1585–605. doi: 10.1007/s10899-024-10287-6 (PMC11390817; doi:10.1007/s10899-024-10287-6)
Supplement: Supplementary file 1 — Supplementary Material 1 [file 10899_2024_10287_MOESM1_ESM.pdf]

Anticipated Motives for Gambling Treatment in Adults from the U.S.

Journal of Gambling Studies

Online Resource 1

Anticipated Motives Questions

Participants were asked the following:

You indicated that you **do not currently think** that you might need treatment or help for your gambling behavior.

Please imagine what it might take or what circumstances might arise that would lead you to believe that you do need such help or treatment. Consider what warning signs, behaviors, or consequences might lead you to think you need help. Keeping that in mind, please answer the following questions:

1. How likely would you be to seek treatment or help for your gambling behavior if:

1. A loved one told you they were concerned about your gambling?
2. A Mental Health Professional told you they thought you had a gambling problem?
3. Your Doctor told you they were concerned about your gambling behavior?
4. You lost more money than you intended on losing?
5. You felt you could not stop gambling?
6. You experienced problems at work because of your gambling?
7. You found yourself lying to conceal your gambling?
8. You felt guilty after gambling?
9. You found yourself needing to bet more and more money?
10. You found yourself in debt to gamble?
11. Your gambling was causing problems in your relationship?
12. You tried to stop gambling but couldn’t?
13. You found yourself gambling to cope with stress or difficult emotions?
14. You found yourself with a strong urge or cravings to gamble?

2. How much money would you have to lose from your gambling to think that you might have a gambling problem? Please answer below by listing how much money you would have to lose in whole numbers only.
