# Supplementary material for: Anticipated Motives for Gambling Treatment in Adults from the U.S
Source: J Gambl Stud. 2024 Feb 24;40(3):1585–605. doi: 10.1007/s10899-024-10287-6 (PMC11390817; doi:10.1007/s10899-024-10287-6)
Supplement: Supplementary file 2 — Supplementary Material 2 [file 10899_2024_10287_MOESM2_ESM.pdf]

Anticipated Motives for Gambling Treatment in Adults from the U.S.

Journal of Gambling Studies

Online Resource 2

Supplementary Figure 1.

Percentage of Participants Engaging In No to Low Problem Gambling Behavior and Moderate to High Problem Gambling Behavior who Endorsed a Loved One Voiced Concerns as an Anticipated Motive

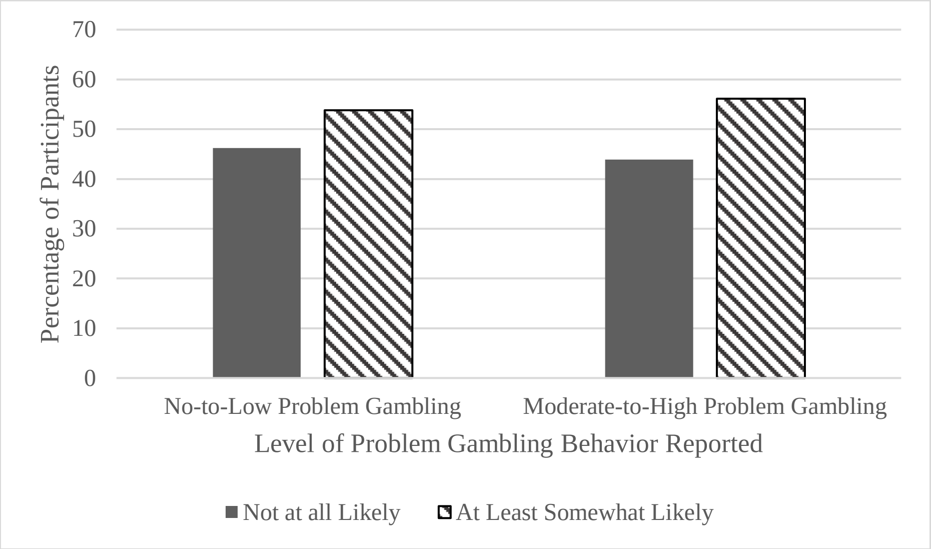

Note: N = 1,791. All analyses shown obtained non-significant χ^2 test result with a *p*-vaue of < .05. Results suggest that there were no significant differences between those who reported moderate-to-high-level problem gambling behavior and those who reported no-to-low-level problem gambling behavior in terms of their endorsement for a Loved One Voiced Concerns as a potential reason to seek treatment.

Supplementary Figure 2.

Percentage of Participants Engaging in No to Low Problem Gambling Behavior and Moderate to High Problem Gambling Behavior who Endorsed a Mental Health Professional Voiced Concerns as an Anticipated Motive

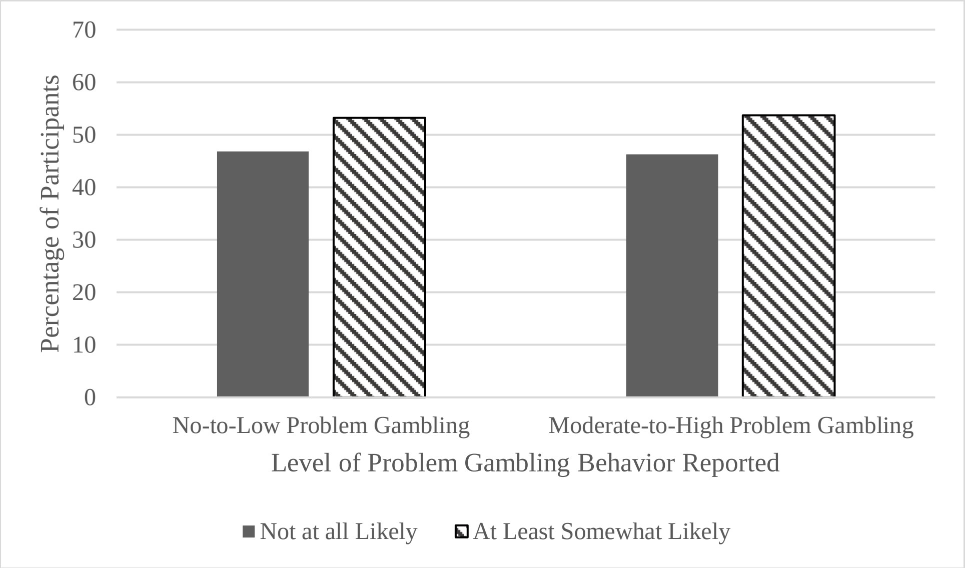

Note: N = 1,791. All analyses shown obtained non-significant χ^2 test result with a p-vaue of < .05. Results suggest that there were no significant differences between those who reported moderate-to-high-level problem gambling behavior and those who reported no-to-low-level problem gambling behavior in terms of their endorsement for a Mental Health Professional Voiced Concerns as a potential reason to seek treatment.

Supplementary Figure 3.

Percentage of Participants Engaging in No to Low Problem Gambling Behavior and Moderate to High Problem Gambling Behavior who Endorsed a Doctor Voiced Concerns as an Anticipated Motive

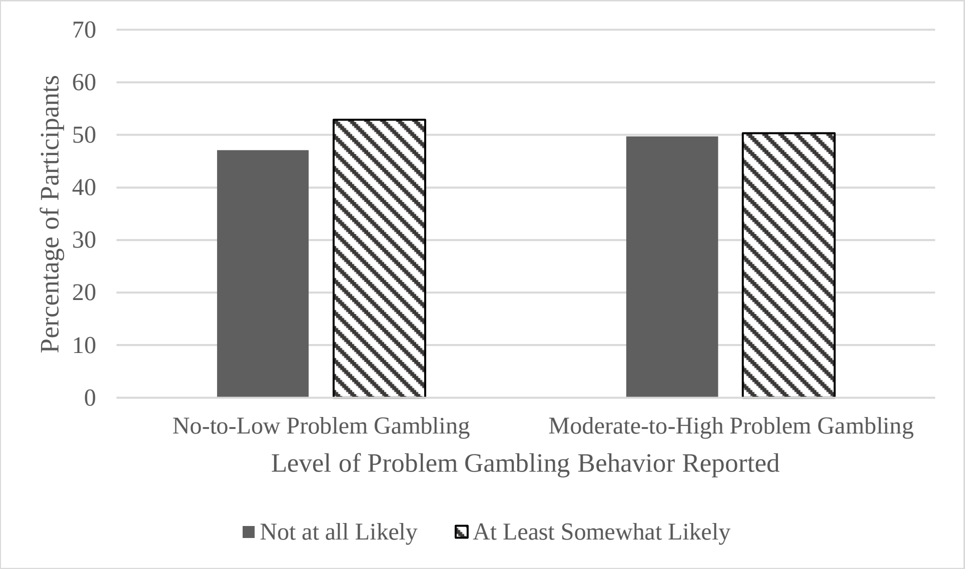

Note: N = 1,791. All analyses shown obtained non-significant χ^2 test result with a *p*-vaue of < .05. Results suggest that there were no significant differences between those who reported moderate-to-high-level problem gambling behavior and those who reported no-to-low-level problem gambling behavior in terms of their endorsement for a Doctor Voiced Concerns as a potential reason to seek treatment.

Supplementary Figure 4.

Percentage of Participants Engaging in No to Low Problem Gambling Behavior and Moderate to High Problem Gambling Behavior who Endorsed Felt You Couldn't Stop as an Anticipated Motive

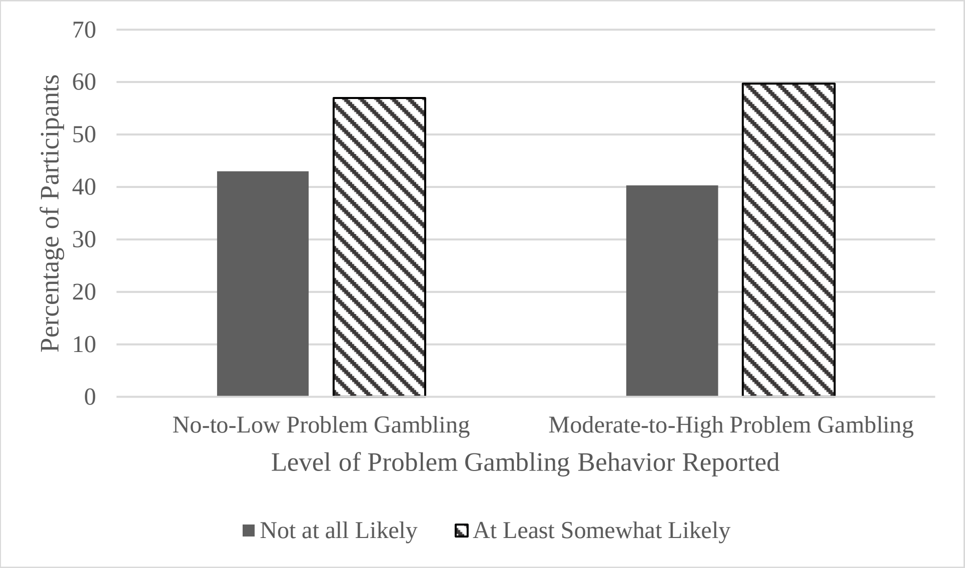

Note: N = 1,791. All analyses shown obtained non-significant χ^2 test result with a *p*-vaue of < .05. Results suggest that there were no significant differences between those who reported moderate-to-high-level problem gambling behavior and those who reported no-to-low-level problem gambling behavior in terms of their endorsement for Felt You Couldn't Stop as a potential reason to seek treatment.

Supplementary Figure 5.

Percentage of Participants Engaging in No to Low Problem Gambling Behavior and Moderate to High Problem Gambling Behavior who Endorsed Work Problems as an Anticipated Motive

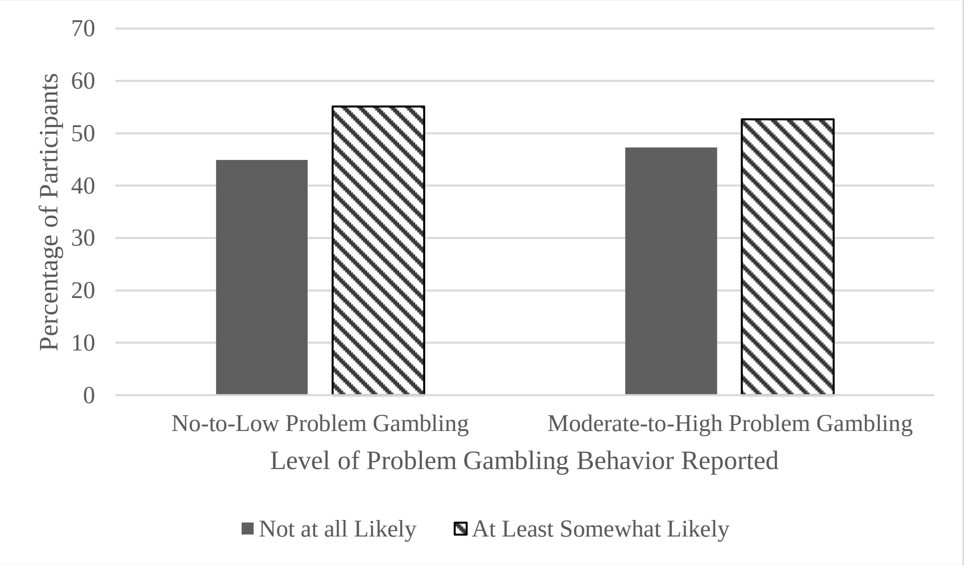

Note: N = 1,791. All analyses shown obtained non-significant χ^2 test result with a *p*-vaue of < .05. Results suggest that there were no significant differences between those who reported moderate-to-high-level problem gambling behavior and those who reported no-to-low-level problem gambling behavior in terms of their endorsement for Work Problems as a potential reason to seek treatment.

Supplementary Figure 6.

Percentage of Participants Engaging in No to Low Problem Gambling Behavior and Moderate to High Problem Gambling Behavior who Endorsed Lying to Conceal Gambling as an Anticipated Motive

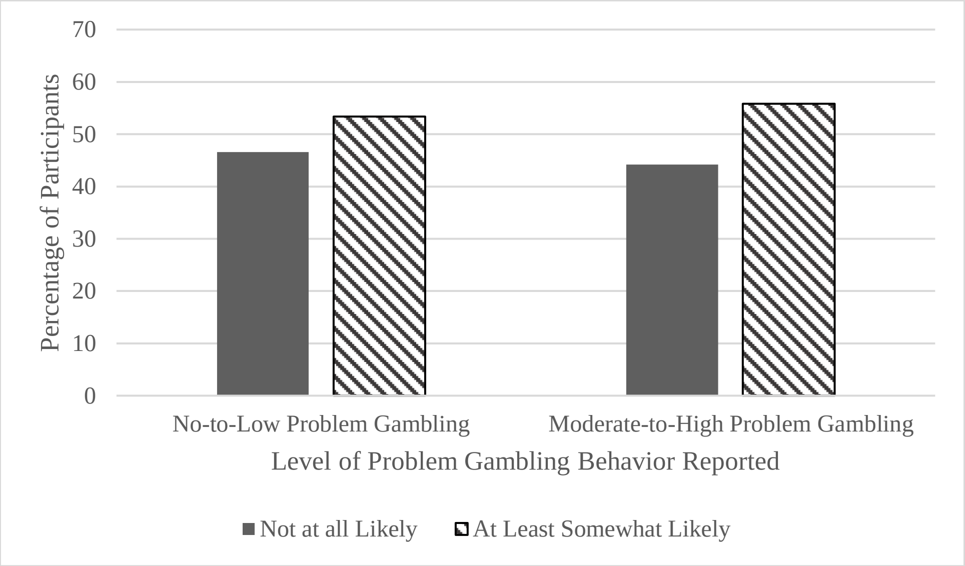

Note: N = 1,791. All analyses shown obtained non-significant χ^2 test result with a *p*-vaue of < .05. Results suggest that there were no significant differences between those who reported moderate-to-high-level problem gambling behavior and those who reported no-to-low-level problem gambling behavior in terms of their endorsement for Lying to Conceal Gambling as a potential reason to seek treatment.

Supplementary Figure 7.

Percentage of Participants Engaging in No to Low Problem Gambling Behavior and Moderate to High Problem Gambling Behavior who Endorsed Needed to Bet More Money as an Anticipated Motive

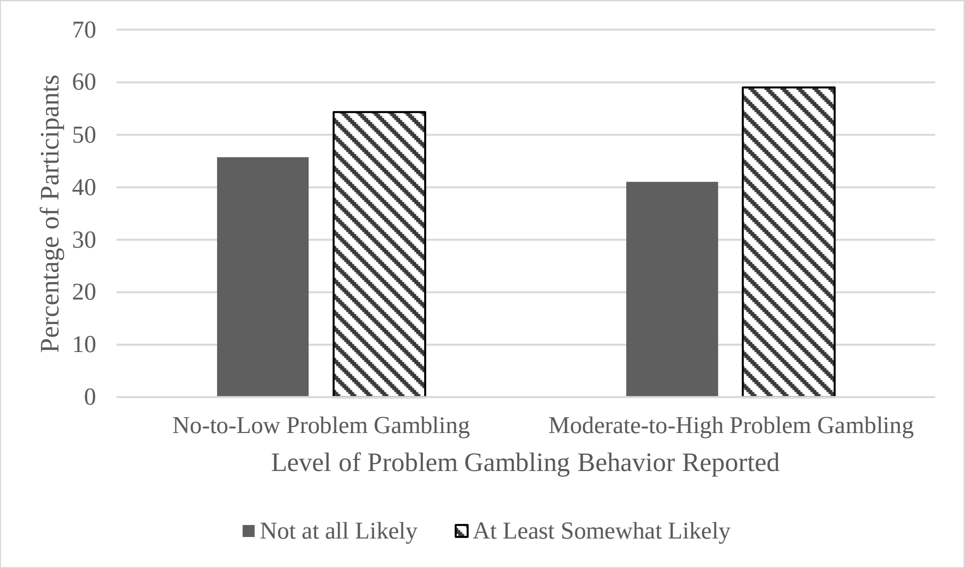

Note: N = 1,791. All analyses shown obtained non-significant χ^2 test result with a p-vaue of < .05. Results suggest that there were no significant differences between those who reported moderate-to-high-level problem gambling behavior and those who reported no-to-low-level problem gambling behavior in terms of their endorsement for Needed to Bet More Money as a potential reason to seek treatment.

Supplementary Figure 8.

Percentage of Participants Engaging in No to Low Problem Gambling Behavior and Moderate to High Problem Gambling Behavior who Endorsed In Debt to Gamble as an Anticipated Motive

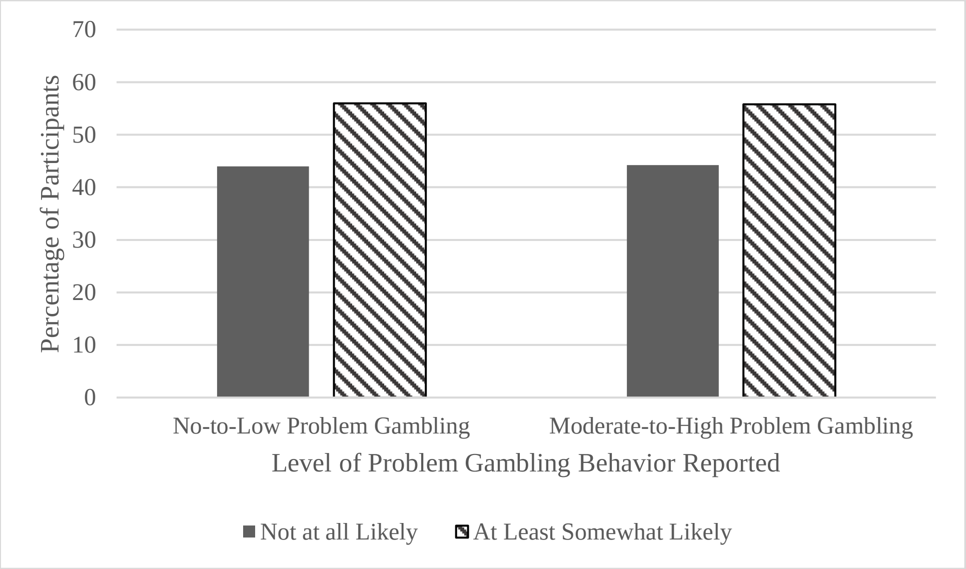

Note: N = 1,791. All analyses shown obtained non-significant χ^2 test result with a *p*-vaue of < .05. Results suggest that there were no significant differences between those who reported moderate-to-high-level problem gambling behavior and those who reported no-to-low-level problem gambling behavior in terms of their endorsement for In Debt to Gamble as a potential reason to seek treatment.

Supplementary Figure 9.

Percentage of Participants Engaging in No to Low Problem Gambling Behavior and Moderate to High Problem Gambling Behavior who Endorsed Tried and Couldn't Stop as an Anticipated Motive

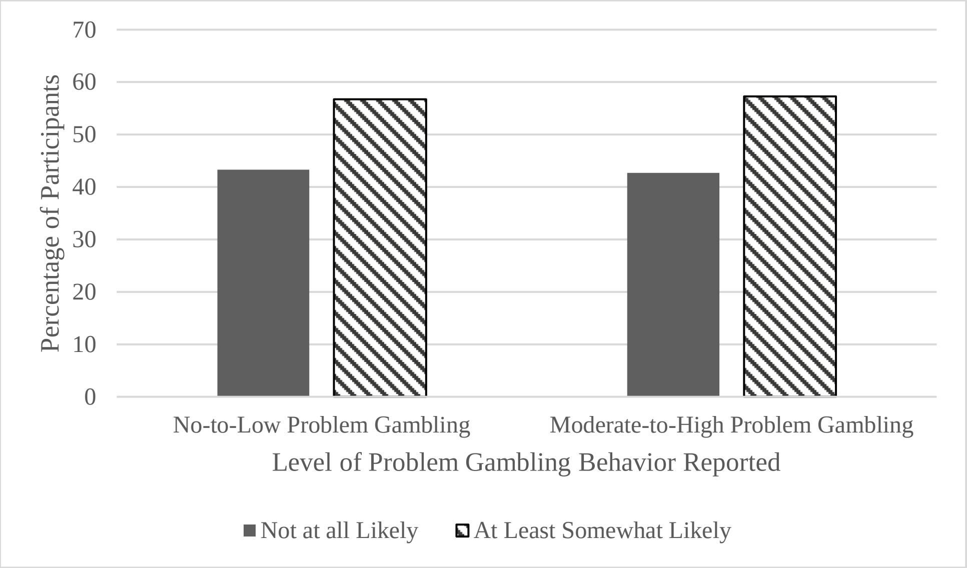

Note: $N = 1{,}791$. All analyses shown obtained non-significant χ^2 test result with a *p*-vaue of $<$ .05. Results suggest that there were no significant differences between those who reported moderate-to-high-level problem gambling behavior and those who reported no-to-low-level problem gambling behavior in terms of their endorsement for Tried and Couldn't Stop as a potential reason to seek treatment.

Supplementary Figure 11.

Percentage of Participants Engaging in No to Low Problem Gambling Behavior and Moderate to High Problem Gambling Behavior who Endorsed Relationship Problems as an Anticipated Motive

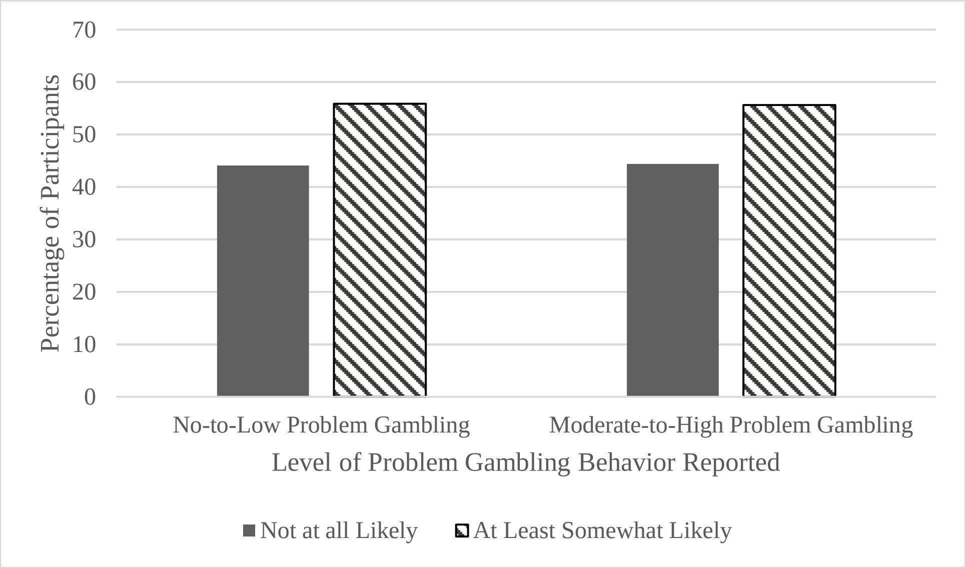

Note: $N = 1{,}791$. All analyses shown obtained non-significant χ^2 test result with a p-vaue of < .05. Results suggest that there were no significant differences between those who reported moderate-to-high-level problem gambling behavior and those who reported no-to-low-level problem gambling behavior in terms of their endorsement for Relationship Problems as a potential reason to seek treatment.
